# The effects of health system reform on medical services utilization and expenditures in China in 2004–2015

**DOI:** 10.1093/inthealth/ihab041

**Published:** 2021-07-14

**Authors:** Zhan Shu, Yingli Liu, Minlin Li, Jian Li

**Affiliations:** School of Public Administration, Central China Normal University, Wuhan, Hubei, 430079, China; School of Public Administration, Central China Normal University, Wuhan, Hubei, 430079, China; School of Public Administration, Central China Normal University, Wuhan, Hubei, 430079, China; Institute of Medical Information, Chinese Academy of Medical Sciences and Peking Union Medical College, Beijing, 100020, China

**Keywords:** China, effects evaluation, health expenditure, health insurance, health reform, medical services utilization

## Abstract

**Background:**

In 2009, China began to implement new health system reform aimed at reducing the financial burden of patients. This study aimed to compare changes in the utilization of medical services and expenditures in different groups from 2004 to 2015.

**Methods:**

Pooled cross-sectional data from 2004 to 2015 from the China Health and Nutrition Survey Database were used to conduct a segmental linear regression to estimate changes in the medical expenditures of different groups before and after implementation of the reform.

**Results:**

During the reform process, the utilization rate of outpatient healthcare, primary health services and hospital health services showed a trend of increasing first and then decreasing slightly. The frequency of residents using inpatient services increased after the reform. The average medical expenditures increased significantly, especially for uninsured and primary health services users.

**Conclusions:**

China's new round of health reform increased the coverage rate of basic medical insurance. Medical insurance has controlled the growth of the average medical expenditures; nevertheless, the average medical expenditures per patient has shown a continuous upward trend. Consequently, both basic medical insurance funds and residents face greater economic burdens and financial risks. Effective methods of controlling the growth of medical expenditures are therefore required.

## Introduction

The new round of health system reform was launched in China in 2009. The goals of the reform were to reduce the financial burden of healthcare, effectively alleviate the high cost of healthcare services and improve the primary healthcare system to provide safe, effective, convenient and inexpensive healthcare services.^1^ In the first 3 y of the reform, from 2009 to 2011, the government invested 1380 billion renminbi (RMB) to improve the health system, including public health services, health insurance, drug supply and healthcare service systems.^2^ To improve the accessibility of healthcare services, the government completed the construction of 29 000 township hospitals. To improve the accessibility and quality of public health services, the national basic public health services project was launched.^3^ To reduce the financial burden, the government provides subsidies to help residents to take advantage of basic medical insurance.^4^

A previous study found that the reform effectively reduced the medical expenditures of inpatients in public hospitals between 2008 and 2011.^5^ However, some studies have shown that the decrease in hospitalization expenditures was limited to out-of-pocket medical expenditures, while the total medical expenditures continued to increase.^6^ In 2012, the Chinese government launched a nationwide reform of county-level public hospitals, with the goal of controlling the rapid growth of healthcare expenditures. The key components of the reform were the zero markup drug policy, which removed the previously allowed 15% markup for drug sales at public hospitals, and associated increases in fees for medical services. Research findings have shown that the policy led to a reduction in drug expenditures, an increase in expenditures for medical testing and diagnostic tests and no measurable changes in total health expenditures.^7^ Expenditures on medicine showed an increasing trend from 2009 to 2013. The average annual growth rate of household overall medical expenditures was significantly higher than that of household non-food consumption expenditures.^8^ Public health institutions were found to be more expensive than private health institutions.^9^

Medical insurance can play an important role in controlling the behaviour of suppliers through payment system optimization. What is the effect of basic medical insurance on medical services expenditures and utilization? Some studies have established that medical insurance is an effective way of reducing inpatient out-of-pocket medical expenditures, but it is difficult to control increases in medical expenditures.^10^ Some studies have shown that total medical and out-of-pocket expenditures are not affected by medical insurance at all.^11,12^ Others have found that basic medical insurance leads residents to use more inpatient care, because the outpatient care had a higher cost-sharing rate.^13,14^ More evidence of the effects of medical insurance on healthcare expenditures and healthcare-seeking choices in China is required.^15^

## Methods

### Study design and data sources

The China Health and Nutrition Survey (CHNS), an ongoing open-cohort international collaborative project between the Carolina Population Center at the University of North Carolina at Chapel Hill and the National Institute for Nutrition and Health at the Chinese Center for Disease Control and Prevention, was designed to examine the effects of the health, nutrition and family planning policies and programs implemented by national and local governments and to see how the social and economic transformation of Chinese society is affecting the health and nutritional status of the population. The survey was conducted using a multistage, random cluster process to produce a sample of about 7200 households with >30 000 individuals in 15 provinces and municipal cities. The CHNS database contains the basic medical insurance type, health service utilization, medical expenditures and other related indicators that can meet the purpose of research. The accuracy, completeness, consistency and reliability of the data has been evaluated. The quality was good. The database was established in 1989 and subsequent surveys and data collection were conducted in 1991, 1993, 1997, 2000, 2004, 2006, 2009, 2011 and 2015. Because the new health system reform in China began in 2009, data from 2004 to 2015 were collected for analysis.

### Procedures

Medical expenditures represented the core indicator of this study and were based on observation of the mean changes in 4-week medical expenditures from 2004 to 2015. Taking 2009 as an intervention point, we analysed the effects of the new health system reform and universal medical insurance policy arrangements on the medical expenditures growth of samples with different medical insurance schemes and different healthcare services. In the regression model, medical expenditures were treated with a base number of 10 for the logarithm.

The types of medical insurance in China include commercial medical insurance, the New Cooperative Medical Scheme (NCMS), Urban Employee Basic Medical Insurance (UEBMI), Gongfei Medical Insurance (GFMI), Urban Resident Basic Medical Insurance (URBMI) and others. Because the proportions of commercial medical insurance and other medical insurance were relatively low, we did not include them in the study. Rather, we compared changes in the medical expenditures of patients with the four basic types of medical insurance and those with no medical insurance.

‘Where did you see a doctor?’ The choice of primary healthcare institute included the village clinic, private clinic, work unit clinic and other clinics and township hospitals. The utilization rates of village clinics and town hospitals were the highest. Hospital care choices included the county maternal and child hospital, county hospital, city maternal and child hospital, city hospital, worker's hospital and other hospitals. Among them, the utilization rates of the city and county hospitals were the highest. The indicators of the utilization of healthcare services included outpatient and inpatient services. Primary healthcare institutes and hospitals provide both outpatient and inpatient services.

Given the major differences in medical expenditures in the different regions of China, it was necessary to take regions as control variables. The eastern regions in the database included Beijing, Shanghai, Jiangsu and Shandong. The central region included Henan, Hunan, Hubei, Heilongjiang and Liaoning. The western region included Guangxi, Guizhou and Chongqing. Family income, age, education level and disease severity were also control variables. Disease severity was assessed through self-evaluation and indicated as one of three levels: not serious, moderate and serious.

### Statistical analysis

Interrupted time series analysis is a quasi-experimental method for evaluating the longitudinal effects of policy interventions.^16^ The segmented time series regression (STSR) model estimated the level and trend of the dependent variable before implementation of the new health system reform and the changes in level and trend after implementation of the reform. The linear regression model was as follows:^17^

$$\begin{eqnarray*}{{\rm{Y}}_{{t}}} &=& {\beta _0} + {\beta _1}{\rm{tim}}{{\rm{e}}_{{t}}} + {\beta _2}{\rm{interventio}}{{\rm{n}}_{{t}}}\nonumber\\
&& +\, {\beta _3}{\rm{time}}\,{\rm{after}}\,{\rm{intervention}} + {\beta _4}{\rm{control}} + {{\rm{e}}_{{t}}}.\end{eqnarray*}$$

Y_*t*_ is the mean value of the evaluation indicator of medical expenditures in year t. Time represents a continuous variable indicating time (in years) at time *t* from the start of the observation period (2004). Intervention represents an indicator for time *t* occurring before (intervention=0) or after (intervention=1) implementation of the reform. The start time of the reform implementation was defined as 2009 and the time after the intervention represents a continuous variable counting the number of years after the intervention at time t, coded 0 before the reform (2004 and 2006) and 1–3 after the reform (2009, 2011 and 2015, respectively). β_0_ estimates the baseline level of medical expenditures at time zero; β_1_ estimates the change in mean value of medical expenditures per year (as a baseline trend per year), namely slope reform;^17^ β_2_ estimates the change in mean value of medical expenditures after the reform (the immediate effect of the reform on medical expenditures); and β_3_ estimates the changes in the trend of the mean value of medical expenditures after the reform compared with the trend before the reform and includes slope change or slope difference. The sum of β_1_ and β_3_ represents the post-reform slope. The error term e_t_ is the random variability not explained by the model. We used Stata software, version 12.0 (StataCorp, College Station, TX, USA).

## Results

China achieved a great breakthrough in the coverage rate of basic medical insurance with the establishment of the new rural cooperative medical insurance system in 2003. By 2009, >50% of citizens had basic medical insurance. The NCMS for rural residents has the largest coverage rate. Among the sample, the proportion of people with GFMI and those without medical insurance has decreased annually. Table 1 shows that the proportion of people covered by basic medical insurance has increased annually.

**Table 1.** Structure of medical health insurance in 2004–2015

Insurance	2004 (n=11 699)	2006 (n=11 386)	2009 (n=11 757)	2011 (n=15 367)	2015 (n=14 966)
NCMS, n (%)	2 861 (24.46)	3199 (28.10)	4133 (35.15)	5423 (35.29)	5870 (39.22)
UEBMI, n (%)	479 (4.09)	597 (5.24)	1029 (8.75)	2686 (17.48)	2589 (17.30)
GFMI, n (%)	835 (7.14)	814 (7.15)	631 (5.37)	639 (4.16)	525 (3.51)
URBMI, n (%)	399 (3.41)	485 (4.26)	830 (7.06)	2014 (13.11)	2089 (13.96)
NBMI, n (%)	7125 (60.90)	6291 (55.25)	5134 (43.67)	4605 (29.97)	3893 (26.01)

NBMI: no basic medical insurance.

The rate of utilization of outpatient services in 4 weeks by residents fluctuated between 7.20% and 19.07%. The rate of utilization of inpatient services by residents increased. The rate of utilization of outpatient services for patients without medical insurance was not lower than that of patients with medical insurance. The rates of utilization of services in primary healthcare institutes of the NCMS and residents without basic medical insurance were higher than that of other residents. In contrast, the rates of utilization of hospital services of the NCMS and residents without basic medical insurance were lower than that of other residents (Table 2).

**Table 2.** Frequency of medical services of residents covered by different medical insurance in 2004–2015

Insurance	2004	2006	2009	2011	2015
Using outpatient services, n (%)					
NCMS	342 (11.95)	332 (10.38)	543 (13.14)	745 (13.74)	631 (10.75)
UEBMI	53 (11.06)	43 (7.20)	94 (9.14)	416 (15.49)	326 (12.59)
GFMI	102 (12.22)	92 (11.30)	73 (11.57)	88 (13.77)	51 (9.71)
URBMI	58 (14.54)	50 (10.31)	98 (11.81)	384 (19.07)	262 (12.54)
NBMI	739 (10.37)	752 (11.95)	631 (12.29)	508 (11.03)	422 (10.84)
Using inpatient services, n (%)					
NCMS	22 (0.77)	20 (0.63)	36 (0.87)	56 (1.03)	79 (1.35)
UEBMI	4 (0.84)	6 (1.01)	18 (1.75)	42 (1.56)	51 (1.97)
GFMI	13 (1.56)	17 (2.09)	14 (2.22)	11 (1.72)	10 (1.90)
URBMI	1 (0.25)	5 (1.03)	7 (0.84)	31 (1.54)	35 (1.68)
NBMI	51 (0.72)	57 (0.91)	55 (1.07)	85 (1.85)	71 (1.82)
Using primary healthcare institute services (outpatient+inpatient), n (%)					
NCMS	267 (9.33)	270 (8.44)	431 (10.43)	607 (11.19)	481 (8.19)
UEBMI	20 (4.18)	13 (2.18)	21 (2.04)	195 (7.26)	157 (6.06)
GFMI	21 (2.51)	25 (3.07)	17 (2.69)	34 (5.32)	26 (4.95)
URBMI	24 (6.02)	25 (5.15)	40 (4.82)	208 (10.33)	144 (6.89)
NBMI	489 (6.86)	498 (7.92)	449 (8.75)	385 (8.36)	314 (8.07)
Using hospital services (outpatient+inpatient), n (%)					
NCMS	86 (3.01)	69 (2.16)	140 (3.39)	181 (3.34)	205 (3.49)
UEBMI	35 (7.31)	33 (5.53)	85 (8.26)	256 (9.53)	206 (7.96)
GFMI	88 (10.54)	79 (9.71)	66 (10.46)	62 (9.70)	34 (6.48)
URBMI	36 (9.02)	28 (5.77)	60 (7.23)	200 (9.93)	143 (6.85)
NBMI	283 (3.97)	257 (4.09)	206 (4.01)	184 (4.00)	165 (4.24)

NBMI: no basic medical insurance.

**Figure 1.**
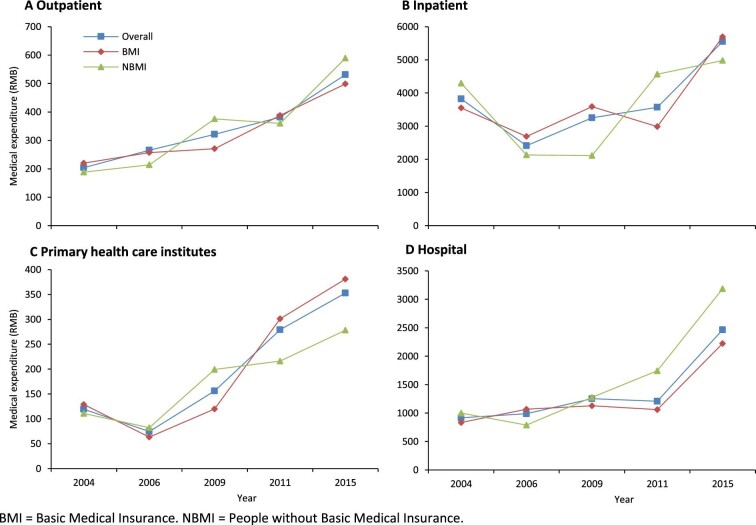
Medical expenditures of patients with insurance and without insurance in 2004–2015.

The medical expenditures of residents covered by the NCMS and of those without basic medical insurance are similar to those of other residents. The medical expenditures of all residents increased (Table 3). The time series of medical expenditures of outpatient and inpatient services from 2004 to 2015 showed a pronounced increasing trend after the health reform. The growth trends of medical expenditures of residents covered by basic medical insurance and those without basic medical insurance were similar. Both primary healthcare services and hospital service medical expenditures increased from 2004 to 2015 (Figure 1).

**Table 3.** Medical expenditures of patients in 2004–2015

Insurance/services	2004	2006	2009	2011	2015
Medical expenditures over 4 weeks per patient (RMB), mean (SD)					
NCMS	728.61 (3313.52)	890.88 (5387.38)	741.51 (2868.04)	1203.69 (5201.19)	2793.29 (13 734.64)
UEBMI	955.10 (2559.49)	1199.78 (4470.21)	2735.27 (6076.99)	2490.44 (8839.40)	3921.84 (12 700.97)
GFMI	1930.16 (8390.42)	1849.61 (6402.73)	2191.60 (5355.19)	1239.47 (2036.84)	6039.11 (12 714.57)
URBMI	643.92 (2618.70)	474.98 (1519.5)	915.57 (3282.99)	1580.69 (6009.18)	2186.06 (13 137.11)
NBMI	929.55 (3657.92)	676.72 (3156.17)	1050.10 (4786.11)	1792.45 (7000.16)	2988.56 (12 099.00)
Medical expenditures over 4 weeks per patient for using services group (RMD), mean (SD)					
Outpatient	442.99 (2679.98)	525.99 (3601.54)	676.37 (3565.60)	893.80 (4318.10)	1474.17 (9372.54)
Inpatient	7192.53 (9580.46)	4607.81 (6414.70)	6332.44 (9298.17)	8004.80 (13 714.75)	13 457.48 (23 441.61)
PHC institute	226.71(974.77)	141.88 (446.33)	316.89 (1551.06)	651.44 (4349.34)	882.45 (5235.75)
Hospital	1849.00 (5793.53)	1942.86 (6530.07)	2547.24 (7176.80)	2907.27 (7495.57)	6318.10 (18 906.54)

NBMI: no basic medical insurance; PHC: primary healthcare.

Based on the STSR results, the medical expenditures of residents covered by the NCMS increased (coefficient=0.169) before 2009 (p>0.1) and showed an immediate increase after the reform (coefficient=0.212, p>0.1). However, they decreased (coefficient=−0.245, p>0.1) before 2009 and increased after the reform (coefficient=0.839, p<0.05) for residents covered by UEBMI. The change in the coefficient of medical expenditures for residents without medical insurance was also clear. It was −0.081 (p>0.1) before 2009 and 0.371 after 2009 (p<0.01). These results show that medical expenditures increased quickly after the reform. The rate of increase in the number of residents without medical insurance was higher than that of the other groups after 2009 (Table 4). This indicates that medical insurance was effective in controlling medical expenditures. The impact of different types of medical insurance on controlling medical expenditures varied before and after the reform. The medical expenditures of patients with UEBMI and URBMI increased significantly in 2009, but the growth of medical expenditures was controlled after 2009. The reason is that medical insurance strengthened the supervision of medical institutions and explored different payment methods.

**Table 4.** Segmental linear regression analysis of medical expenditures of patients covered by different medical insurance in 2004–2015

Variable	NCMS	UEBMI	GFMI	URBMI	NBMI
β_0_	0.750** (0.378)	3.722*** (0.838)	1.207 (0.744)	2.442* (1.243)	1.251*** (0.347)
β_1_ time (slope pre-reform)	0.169 (0.141)	−0.245 (0.387)	0.073 (0.34)	−0.385 (0.295)	−0.081 (0.099)
β_2_ intervention (level change after reform)	0.212 (0.159)	0.839** (0.384)	0.643* (0.358)	0.882** (0.426)	0.371*** (0.14)
β_3_ time (slope change after reform)	0.056 (0.153)	0.282 (0.402)	0.078 (0.359)	0.472 (0.347)	0.335*** (0.116)
β_1_+β_3_ (slope post-reform)	0.225	0.037	0.151	0.087	0.254
Log(household income)	0.086*** (0.033)	−0.071 (0.071)	0.095 (0.061)	0.049 (0.112)	0.086*** (0.031)
Age	0.013*** (0.002)	0.014*** (0.005)	0.004 (0.004)	0.013* (0.007)	0.012*** (0.002)
Education	0.121*** (0.038)	0.034 (0.05)	0.065 (0.05)	−0.054 (0.06)	0.132*** (0.032)
Severity of disease	1.147*** (0.059)	0.878*** (0.106)	1.163*** (0.111)	1.081*** (0.162)	1.188*** (0.052)
Central area vs east area	0.146 (0.097)	0.466*** (0.167)	0.0607 (0.186)	0.274 (0.238)	−0.157 (0.102)
West area vs east area	−0.286*** (0.1)	0.071 (0.198)	−0.091 (0.188)	−0.808*** (0.269)	−0.765*** (0.108)
R^2^	0.233	0.152	0.243	0.243	0.258
Sample size, n	1927	641	527	313	2480

NBMI: no basic medical insurance.

The values in parentheses are standard errors.

*p<0.1, **p<0.05, ***p<0.01.

The medical expenditures of outpatients did not increase noticeably before 2009 (Table 5). Expenditures of outpatients showed an immediate increase after the reform (coefficient=0.440 and 0.474, p<0.01). The medical expenditures of inpatients decreased (coefficient=−0.368 and −0.337, p>0.1) before 2009 and also showed an immediate decrease (coefficient=−0.140 and −0.521, p>0.1) after initiation of the reform. There was rapid growth after 2009 (coefficient=0.681 and 0.844, p<0.01). The data indicate that both outpatient and inpatient expenditures increased significantly. The medical expenditures for primary healthcare showed no significant increase before 2009 but showed a significant increase after the reform (coefficient=0.356 and 0.628, p<0.01). The increase in hospital care was not significant before the reform (coefficient=0.064, p>0.1), but the increasing trend was significant after 2009 (coefficient=0.527, p<0.01). In 2009, the medical expenditures of outpatient and primary healthcare services increased significantly for all patients, but the medical expenditures of inpatient and hospital services did not increase. However, from 2009 to 2014, medical expenditures of hospitalization showed a significant growth trend in primary healthcare institutes and general hospitals and the growth of expenditures was more significant for patients without basic medical insurance.

**Table 5.** Segmental linear regression analysis of medical expenditures of patients by service utilization in 2004–2015

Variables	Outpatient		Inpatient		PHC		Hospital	
	BMI	NBMI	BMI	NBMI	BMI	NBMI	BMI	NBMI
β_0_	1.299*** (0.267)	1.616*** (0.330)	6.231*** (0.792)	5.805*** (0.981)	1.204*** (0.304)	1.607*** (0.357)	4.024*** (0.466)	4.130*** (0.612)
β_1_ time (slope pre-reform)	0.060 (0.097)	−0.057 (0.092)	−0.368 (0.321)	−0.337 (0.327)	0.046 (0.117)	−0.100 (0.103)	0.174 (0.156)	−0.019 (0.164)
β_2_ intervention (level change after reform)	0.440*** (0.115)	0.474*** (0.132)	−0.140 (0.341)	−0.521 (0.407)	0.356*** (0.134)	0.628*** (0.143)	0.263 (0.185)	−0.004 (0.242)
β_3_ time (slope change after reform)	0.037 (0.106)	0.164 (0.109)	0.681** (0.340)	0.844** (0.363)	0.142 (0.127)	0.236** (0.120)	0.064 (0.170)	0.527*** (0.194)
β_1_+β_3_ (slope post-reform)	0.097	0.107	0.313	0.517	0.188	0.136	0.238	0.508
Log(household income)	0.112*** (0.023)	0.080*** (0.029)	−0.131* (0.070)	0.075 (0.082)	0.078*** (0.027)	0.064** (0.031)	−0.086** (0.040)	−0.022 (0.055)
Age	0.013*** (0.001)	0.011*** (0.002)	0.016*** (0.005)	0.003 (0.006)	0.013*** (0.002)	0.011*** (0.002)	0.014*** (0.002)	0.005 (0.003)
Education	0.127*** (0.019)	0.133*** (0.030)	0.192*** (0.055)	0.242*** (0.088)	0.107*** (0.025)	0.099*** (0.035)	−0.023 (0.027)	0.006 (0.049)
Severity of disease	0.768*** (0.042)	0.906*** (0.051)	0.980*** (0.123)	0.620*** (0.143)	0.763*** (0.050)	0.811*** (0.059)	0.958*** (0.066)	0.956*** (0.084)
Central area vs east area	0.050 (0.064)	−0.155 (0.097)	−0.435** (0.177)	−0.465* (0.265)	0.064 (0.077)	−0.189* (0.107)	0.269*** (0.099)	−0.118 (0.165)
West area vs east area	−0.401*** (0.069)	−0.686*** (0.102)	−0.529** (0.211)	−0.656** (0.296)	−0.303*** (0.081)	−0.698*** (0.112)	−0.356*** (0.112)	−0.573*** (0.180)
R^2^	0.196	0.203	0.231	0.165	0.211	0.219	0.224	0.227
Sample size, n	3390	2233	370	246	2141	1616	1538	785

BMI: basic medical insurance; NBMI: no basic medical insurance.

The values in parentheses are standard errors.

*p<0.1, **p<0.05, ***p<0.01.

## Discussion

In China, the new health system reform launched in 2009 focused on reforming the public hospital system, improving the coverage of medical insurance and strengthening primary care institutes. The government thought that such policies would reduce the financial burden of healthcare and provide safe, effective, convenient and inexpensive healthcare services. In the past decade, the main achievements of China's health reform have been the establishment of universal health insurance through a multilevel health insurance system with wide coverage and increasing investment in primary medical institutions. However, there is a need to understand how the reform controls medical expenditures and changes the utilization of medical services. This study analysed the effects of the reform.

One key measure of the reform was improvements in residents’ use of primary healthcare to save money. Strengthening primary healthcare services, a key point of the reform, was done by increasing investment in primary healthcare to improve equipment, cooperation between hospitals and building a medical association union to improve the primary healthcare service level. The study found that the utilization rate of primary healthcare services increased widely from 2004 to 2011; however, it decreased from 2011 to 2015. These changes may be related to policies that encourage hospitals to decrease outpatient services for chronic diseases and transfer patients to primary healthcare institutes and encourage the primary health institutes to provide public services. The services of primary healthcare institutes for providing long-term prescriptions to older patients have decreased in recent years. The frequency of residents visiting doctors has decreased. The rate of use of outpatient services in all groups showed an increase in the first period and a decrease after 2011, possibly because some outpatient services had no or low compensation from medical insurance. This may have led some outpatient services to convert to inpatient services to receive more compensation from insurance. There is also a significant correlation between the investment in primary healthcare institutes and the increase in medical expenditures of primary healthcare,^18^ which could be important, as it would make the total medical expenditures increase quickly.

Strengthening medical insurance payment reform to control medical expenditures was also an important measure. China has started to move away from the fee-for-service payment model to experiment with alternative payment plans.^6^ Payment by diagnosis-related groups or diseases combined with clinical pathways is used in many regions.^19^ These methods may control the average expenditures per time; however, if providers split the services, it can potentially lead to overdiagnosis and the total expenditures in a certain period will not be controlled. This study found a growth trend in the average medical expenditures per patient over 4 weeks among the different medical insurance groups. For UEBMI, URBMI and those without medical insurance, medical expenditures were effectively controlled before the reform but exhibited a growth trend after the reform. For the NCMS and GFMI, medical expenditures increased before and after the reform.

In addition, this study found a similar increasing rate of inpatient and outpatient expenditures and frequency of use for residents with basic medical insurance and those not covered by medical insurance, meaning that the total medical expenditures increased after the reform. Residents who had no basic medical insurance spent more money on medical services, especially inpatient services. This result shows that basic medical insurance played a role in controlling the growth of medical services expenditures and in encouraging residents to use primary healthcare after 2009. In recent years, most reform policies have focused on decreasing the cost of public hospitals, but hospital costs are still increasing at a rapid rate. The effect of medical insurance in controlling the growth of hospitalization expenditures is not significant. Medical insurance should pay more attention to controlling medical expenditures.

After 2015, a series of changes occurred in China's medical services system, including implementation of the community family doctor signing service, the construction of medical consortia, integration of the medical insurance system, the adoption of value-based strategic purchasing in the global budget and the establishment of a long-term care insurance system. How these policies affect the growth of medical expenditures in different medical services will require further observation.

This study produced a simple evaluation, that the increase in medical expenditures and utilization of services is affected by many factors. It is difficult to say whether the reform has been effective or not. The limitations of this study include the lack of evaluation of the impact of healthcare quality, technological innovation and other factors on the growth of medical expenditures as well as the impact of healthcare system reform on the healthcare quality. In the future, research should analyse expenditures in outpatient and inpatient services and the factors affecting medical institutes to help the government find the keys to controlling medical expenditures. Furthermore, the government has issued a series of policies to improve the medical system and a long-term observation study is necessary to evaluate the effects of these reforms.

## Conclusions

This study analysed the changes in medical expenditures and utilization of residents before and after healthcare system reform. Medical insurance has controlled the growth of average medical expenditures, but the average medical expenditures per patient shows a continuous upward trend. Both basic medical insurance funds and residents may face greater economic burdens or financial risks. Both primary and public healthcare may be the key to controlling medical expenditures. Reforms should strengthen the primary and public healthcare system so residents can get earlier and better health interventions and health insurance should pay more attention to preventing the incidence of diseases in China.

## Data Availability

Data underlying this article were obtained from the China Health and Nutrition Survey website (https://www.cpc.unc.edu/projects/china).
